# Specificity of the Receptor for the Major Sex Pheromone Component in *Heliothis virescens*

**DOI:** 10.1673/031.013.16001

**Published:** 2013-12-25

**Authors:** Gissella M. Vásquez, Zainulabeuddin Syed, Patricia A. Estes, Walter S. Leal, Fred Gould

**Affiliations:** 1 Department of Entomology and W. M. Keck Center for Behavioral Biology, North Carolina State University, Raleigh, NC 27695, USA; 2 Department of Biological Sciences, University of Notre Dame, Notre Dame, IN 46556, USA; 3 Department of Biological Sciences and W. M. Keck Center for Behavioral Biology, North Carolina State University, Raleigh, NC 27695, USA; 4 Department of Entomology, Honorary Maeda-Duffey Laboratory, University of California, Davis, CA 95616, USA

**Keywords:** *Drosophila melanogaster*, HvORI3, T1 sensilla

## Abstract

In a previous study, the *Drosophila melanogaster OR67d^GAL4^;UAS* system was used to functionally characterize the receptor for the major component of the sex pheromone in the tobacco budworm, *Heliothis virescens* Fabricius (Lepidoptera: Noctuidae), HvOR13. Electrophysiological and behavioral assays showed that transgenic flies expressing HvOR13 responded to (Z)-11-hexadecenal (Z11-16:Ald). However, tests were not performed to determine whether these flies would also respond to secondary components of the *H. virescens* sex pheromone. Thus, in this study the response spectrum of HvOR13 expressed in this system was examined by performing single cell recordings from odor receptor neuron in trichoid T1 sensilla on antennae of two *Or67d*^GAL4 [1]^; *UAS-HvOR13* lines stimulated with Z11-16:Ald and six *H. virescens* secondary pheromone components. Fly courtship assays were also performed to examine the behavioral response of the *Or67d*^GAL4[1]^; *UAS-HvOR13* flies to Z11-16:Ald and the secondary component Z9-14:Ald. Our combined electrophysiological and behavioral studies indicated high specificity and sensitivity of HvOR13 to Z11-16:Ald. Interestingly, a mutation leading to truncation in the HvOR13 C-terminal region affected but did not abolish pheromone receptor response to Z11-16:Ald. The findings are assessed in relationship to other HvOR13 heterologous expression studies, and the role of the C-terminal domain in receptor function is discussed. A third line expressing HvOR15 was also tested but did not respond to any of the seven pheromone components.

## Introduction

Studies on pheromone processing by male moths have greatly contributed to the understanding of the mechanisms involved in animal sensory perception (Rützler and Zwiebel 2005; Touhara and Vosshall 2009; Kaupp 2010). Work in this area has been focused mainly on two moth species, *Bombyx mori* and *Heliothis virescens* Fabricius (Lepidoptera: Noctuidae), with the former having a simple two-component pheromone blend (Kaissling and Kasang 1978) and the latter a more complex pheromone blend (Vetter and Baker 1983). Molecular aspects of male pheromone reception in these two moths have been examined using different approaches (Sakurai et al. 2004, 2011; Nakagawa et al. 2005; Grosse-Wilde et al. 2007; Kurtovic et al. 2007) for which differences in the level of pheromone receptor response specificity and sensitivity have been observed.

The *GAL4/UAS* targeted gene expression system in *Drosophila melanogaster* (Brand and Perrimon 1993) has been used to functionally characterize odorant receptors (ORs) in insects (Dobritsa et al. 2003; Hallem et al. 2004a; Jones et al. 2005; Carey et al. 2010). The “empty neuron” in the ab3 basiconic sensilla (▵halo;*OR22a-Gal4/UAS)* has been a useful tool for characterization of general odorant receptors as well as sex pheromone receptors (Hallem et al. 2004b; Hallem and Carlson 2006; Syed et al. 2006), while the knock-in mutant in trichoid T1 sensilla *(OR67d*^GAL4^;*UAS)* has been used to functionally characterize the *D. melanogaster* receptor for the pheromone cVA (Z11-18:OAc) (Ha and Smith 2006) and two moth sex pheromone receptors (Kurtovic et al. 2007). Recently, Syed et al. (2010) expressed a pheromone receptor from the silkworm moth, *B. mori, BmORl*, in both of these expression systems (i.e., basiconic and trichoid) and found a more sensitive and specific receptor response in fly trichoid sensilla Tl than in the ab3 basiconic sensilla. The latter system not only needed higher doses of bombykol to stimulate the receptor, but was also unusually sustained, suggesting that this system is less suitable for testing pheromone receptors.

In male moths, neurons expressing pheromone receptors are housed in trichoid sensilla dedicated to pheromone reception (Christensen and Hildebrand 2002). Therefore, the higher sensitivity and specificity of the *D. melanogaster* trichoid Tl sensilla system expressing *BmORl* may be due to the innate biochemical machinery and structural features of sensilla for detection of the sex pheromones, in which case a similarly sensitive and specific response is expected for other moth sex pheromone receptors. For example, the *H. virescens* major pheromone component receptor, HvOR13, expressed in *D. melanogaster* trichoid Tl sensilla was found to respond to its putative ligand (Z)-11-hexadecenal (Z11-16:Ald) (Kurtovic et al. 2007). However, it is unclear whether this receptor, expressed in *D. melanogaster,* has high specificity for Z11-16:Ald or could also respond to some or all of the secondary components of the *H. virescens* sex pheromone. Functional analyses of other moth pheromone receptors by Wanner et al. (2010) and Miura et al. (2010) both found that some pheromone receptors of *Ostrinia nubilalis* were broadly tuned, while one receptor appeared to be highly specific for one pheromone component. Our study was therefore designed to determine the degree of specificity of HvOR13 expressed in this system by performing single cell recordings from odor receptor neurons in trichoid Tl sensilla on antennae of two *Or67d*^GAL4 [1]^; *UAS-HvORl3* lines and a control line (*Or67d*^+[1]^) stimulated with Z11-16:Ald and six *H. virescens* secondary pheromone components. The electrophysiological response of another construct, *Or67d*^GAL4 [1]^;*UAS-HvOR15,* to the seven *H. virescens* pheromone components was also examined. HvOR15 was considered a candidate receptor for Z9-14:Ald (Baker 2009; Krieger et al. 2009, Gould et al. 2010); however, it has been shown that it does not respond to this pheromone component in a *Xenopus laevis* oocyte system (Wang et al. 2011), a finding that we expect to corroborate in the *D. melanogaster* trichoid Tl sensilla system. In addition, fly courtship assays were performed to examine the behavioral response of the *Or67d*^GALA [1]^;*UAS-HvORl3* flies to Z11-16:Ald and Z9-14:Ald. Expression levels of *HvOR13* in both *Or67d*^GAL4 [1]^; *UAS-HvOR13* lines were measured by qRT-PCR to determine if *HvOR13* expression was associated to differential electrophysiological and behavioral responses between the two *Or67d*^GAL4 [1]^; *UAS-HvORl3* lines. The combined electrophysiological and behavioral studies indicated that HvOR13 showed high specificity and sensitivity for Z11-16:Ald, with results comparable to those observed for HvOR13 heterologously expressed in *X. laevis* oocytes (Wang et al. 2011), a useful finding considering that expression of other insect odorant receptors in these two systems do not always produce similar results (Carey et al. 2010; Wang et al. 2010). Interestingly, a truncation of the HvOR13 C-terminal region appeared to affect, but did not completely abolish, pheromone receptor function, a finding that could be linked to the functional importance of the C-terminal domain in the formation of the odorant receptor/Orco heteromeric complex (Benton et al. 2006; de Bruyne et al. 2009; Vosshall and Hansson 2011).

## Materials and Methods

### *Drosophila melanogaster* stocks

**Figure 1.**
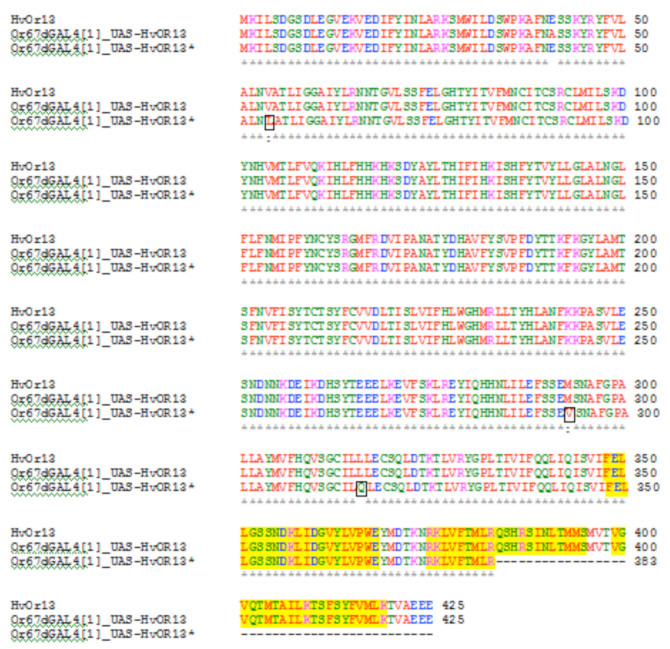
Alignment of HvOR13 translated sequences from *Heliothis virescens* and *Drosophila melanogaster Or67d*^GAL4[1]^;*UAS-HvOR13* lines. Conserved and major amino acid changes in *Or67d*^GAL4[1]^;UAS-HvOR13* are boxed in black. Motifs A, B and C in the C-terminal region of HvOR13 are highlighted in yellow. High quality figures are available online.

*Drosophila melanogaster* stocks *Or67d*^+[1]^ *Or67d*^GAL4 [1]^, and *Or67d*^GAL4[1]^;*UAS-HvOR13* (Kurtovic et al. 2007) were provided by A. Widmer, Research Institute of Molecular Pathology (Vienna, Austria). UAS-HvOR13* and *UAS-HvOR15* were prepared by subcloning an H. virescens OR13* cDNA and an *H. virescens OR15* cDNA, respectively, into pUAS (donated by J. Mahaffey, North Carolina State University, Department of Biological Sciences, Raleigh, NC) using Novagen pSTBlue-1 Acceptor Vector Kit (EMD Millipore, www.emdmillipore.com). The HvOR13* cDNA contained two amino acid polymorphisms and an early termination codon (Figure 1). Transgenic fly lines containing UAS-HvOR13* and *UAS-HvOR15* were generated by Rainbow Transgenic Flies, Inc. (www.rainbowgene.com). To express the HvOR13* and HvOR15 in the trichoid Tl sensilla, six UAS-HvOR13* lines were crossed to *Or67d*^GAL4 [1]^ and four *UAS-HvOR15* lines were crossed to *Or67d*^GAL4 [1]^.

### Single sensillum recordings

Recordings were performed as described by Syed et al. (2006). In brief, a *D. melanogaster* adult was restrained, a glass reference electrode was placed in the eye, and the recording electrode was brought into contact with the base of the trichoid sensillum. Recorded extracellular action potentials (spontaneous and upon stimulation) were amplified, fed into an IDAC4-USB box (Syntech, www.syntech.nl), recorded, and analyzed with Auto Spike version 3.7 (Syntech). AC signals (action potentials or spikes) were band-pass filtered between 100 and 10,000 Hz. The preparation was held in a humidified air stream delivered at 20 mL/sec, to which a stimulus pulse of 2 mL/sec was added for 500 msec. Unless specified otherwise, signals were recorded for 10 sec starting 2 sec before stimulation, and spikes were counted off-line in a 500 msec period before and during the stimulation. Responses from individual neurons were calculated as the increase in spike frequency (spikes/sec) relative to the pre-stimulus frequency. At least three flies of each of the four genotypes (*Or67d*^+[1]^, *Or67d*^GAL4 [1]^; *UAS-HvOR13, Or67d*^GAL4 [1]^;UAS-HvORl3*, and *Or67d*^GAL4 [1]^*\UAS-HvORl5)* were examined, and recordings were made from up to five sensilla from each fly tested. Data were pooled after observing no significant differences between sensilla, sexes, or age groups (1- to 5-day-old flies) were observed.

The following compounds were used as stimuli: (Z)-ll-hexadecenal (Z11-16:Ald), (Z)-9-tetradecenal (Z9-14:Ald), (Z)-9-hexadecenal (Z9-16:Ald), hexadecanal (16:Ald), (Z)-11-hexadecenyl acetate (Z11-16:OAc), (Z)-11-hexadecen-1-ol (Z11-16:OH), and (Z)-9-hexadecen-l-ol (Z9-16:OH) (all 93–95% minimum purity) and cVA ( ⩾98% purity) . All compounds were purchased from Bedoukian (www.bedoukian.com) except for cVA, which was purchased from Cayman Chemical (www.caymanchem.com). A 10 μL aliquot of a stimulus dissolved in double distilled hexane at the desired dose was loaded on a filter paper strip, the solvent evaporated for 30 sec, and the strip was placed in a disposable Pasteur pipette. Hexane loaded on a filter paper strip alone and an empty Pasteur pipette served as controls. All pheromone components were tested at 10 μg source dose for initial screening and subsequent recordings were performed with varying doses ranging from 10 ng to 10 μg. Source doses indicated throughout the manuscript for electrophysiology data represent the dilutions of the solution deposited onto the filter paper of the stimulus cartridge. Thus, a source dose of 10 μg means a 10 μL solution of 10 μg/μL was deposited.

### Courtship assays

Single pair courtship assays were performed following Kurtovic et al. (2007). In brief, *Or67d*^+[1]^ virgin female flies (5–8 days old) were treated by applying 0.2 μL of Z11-16:Ald (4 μg), Z9-14:Ald (2 μg), or acetone (solvent control) onto their dorsal abdomens while lightly anaesthetized with CO_2_. Treated females were placed individually in a round chamber (10 mm diameter and 4 mm height) with moistened nitrocellulose paper and paired with single *Or67d*^+[1]^, *Or67d*^GAL4 [1]^;*UAS-HvORl3*, or *Or67d*^GAL4 [1]^;UAS-HvOR13* male flies (5*8 days old). A clear acetate sheet prevented contact between female and male flies. Flies were allowed to recover for 1 hr before behavioral assays were performed. Courtship index, the percentage of time for which the male courts the female during a 10-min assay, was used to quantify male courtship behavior. In these assays, *Or67d*^+[1]^ male flies were expected to be avid courters in greater than 70% of all treated female flies. In the courtship ritual, the male orients toward and follows the female, taps her with his forelegs, sings a courtship song by extending and vibrating one wing, licks her genitalia, and finally curls his abdomen for copulation (Demir and Dickson 2005). In contrast, *Or67d*^GAL4 [1]^; *UAS-HvOR13* and *Or67d*^GAL4 [1]^;UAS-HvORl3* male flies were expected to display a reduced courtship index when paired with females treated with Z11-16:Ald, as this compound would inhibit male courtship behavior in these transgenic flies, which have HvOR13 replacing Or67d, the receptor for cVA, a male sex pheromone that inhibits male courtship.

### *HvOR13* expression levels by qRT-PCR

*HvOR13* mRNA expression levels were measured in heads of *Or67d*^GAL4 [1]^;UAS-HvOR13 and *Or67d*^GAL4 [1]^;UAS-HvORl3* male flies by qRT-PCR. Total RNA from each sample (pool of five adult heads) was isolated and purified using the RNeasy Plus Mini Kit (Qiagen, www.qiagen.com). RNA quality and quantity were determined using a NanoDrop ND-1000 Spectrophotometer (NanoDrop Technologies, www.nanodrop.com). Total RNA (150 ng) was converted to cDNA using Ambion ArrayScript reverse transcriptase (Ambion, Invitrogen, www.invitrogen.com). Random hexamers (Applied Biosystems, Invitrogen) were used as cDNA synthesis primers in a reaction mix that included 10X Array Script buffer (Ambion), RNAseOUT (Invitrogen), and 10 mM dNTP (Invitrogen). Primers targeting exons of *HvOR13* and the housekeeping gene *RP49* were designed with PRIMER EXPRESS 2.0 software (Applied Biosystems) set to select for an optimal primer annealing temperature of 59° C (58–60° C), amplicon sizes of 40–150 bp, a -3′GC clamp = 0, and a minimum and maximum GC content of 30% and 80%, respectively. Primers were designed based on *H. virescens OR13* and *B. mori RP49* mRNA sequences obtained from GenBank database. Quantitative RT-PCR was performed with an ABI Prism 7900 sequence detector and 96-well optical reaction plates (Applied Biosystems). All reactions were performed in triplicate in a total volume of 10 μL containing 5 μL of SYBR Green PCR Master Mix (Applied Biosystems) and 10 μM of each primer under the following conditions: 50° C for 2 min, 95° C for 10 min followed by 40 cycles of denaturation at 95° C for 15 sec, annealing and extension at 60° C for 1 min, followed by 95° C for 15 sec and 60° C for 15 sec. A dissociation curve and negative control (cDNA reaction without reverse transcriptase enzyme) were used to ensure primer specificity and lack of contamination. Six samples per genotype were examined, and the same samples were run on separate plates twice (two runs). A standard curve was generated for each primer set using dilutions of genomic DNA to calculate the relative quantities of mRNA levels. For each sample, the ratio of the expression level of the target gene to that of the control gene *(RP49)* was calculated (ABI User Bulletin 2, Applied Biosystems) and used for data analysis.

### *HvOR13* cDNA sequence comparison between *Or67d*^GAL4[1]^*;UAS-HvOR13* lines

*HvOR13* was amplified by RT-PCR from *H. virescens* male antennae and from heads of *Or67d*^GAL4 [1]^; *UAS-HvOR13* and *Or67d*^GAL4 [1]^;UAS-HvORl3* flies using gene specific primers designed based on published *H. virescens* cDNA sequences (Krieger et al. 2004). *H. virescens* male antennal RNA, and RNA from heads of *Or67d*^GAL4 [1]^;UAS-HvOR13 and *Or67d*^GAL4 [1]^;UAS-HvOR13* lines, were isolated using Qiagen RNeasy Plus. cDNA was synthesized using Qiagen QuantiTect Reverse Transcription kit with a total RNA concentration of 0.5 μg. PCR was performed using the FastStart High-fidelity PCR system (Roche, www.roche.com) under the following conditions: 94° C for 3 min followed by 19 cycles of denaturation at 94° C for 1 min, annealing at 57° C for 1 min with 0.5° C decreasing per cycle, extension at 72° C for 2 min followed by 19 cycles of denaturation at 94° C for 1 min, annealing at 47° C for 1 min and extension at 72° C for 2 min, followed by 72° C for 7 min. PCR products were purified and sequenced by Genewiz (www.genewiz.com). Nucleotide and translated sequences were aligned using ClustalW2 (EMBL-EBI, www.ebi.ac.uk).

## Results

### Electrophysiological assays

**Figure 2.**
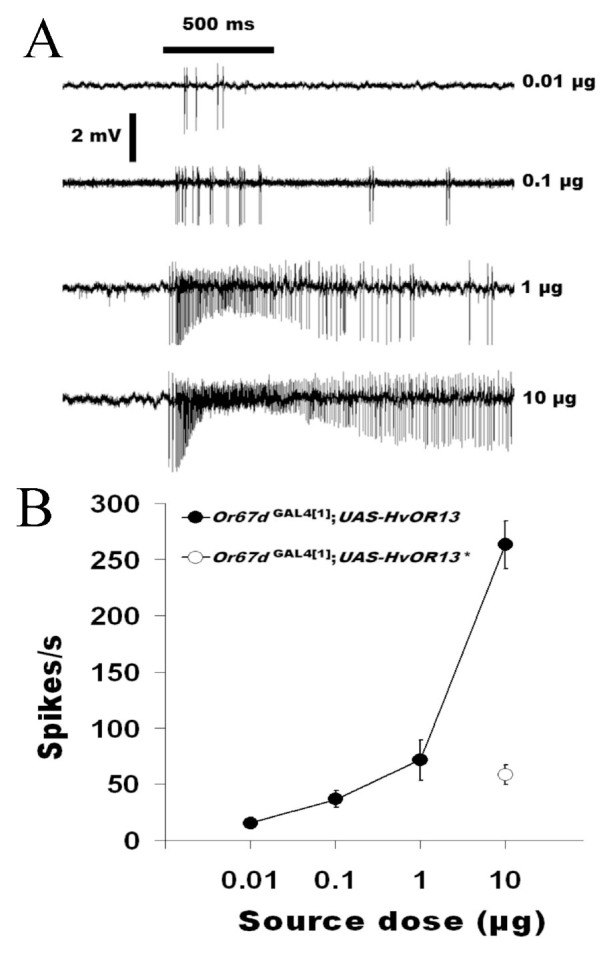
A major component of *Heliothis virescens* pheromone Z11-16:Ald induces dose dependent excitatory responses from an olfactory receptor neuron expressing the *H. virescens* moth pheromone receptor HvORl3 in a trichoid sensillum of *Drosophila melanogaster Or67d*^GAL4[1]^*;UAS-HvOR13* but not from one expressing HvORl3* in a trichoid sensillum of *D. melanogaster Or67d*^GAL4[1]^;UAS-HvOR13*. A. Traces of the excitatory responses recorded from *Or67d*^GAL4[1]^;*UAS-HvOR13* Tl sensilla to increasing pheromone dose. B. Dose-response curve for *Or67d*^GAL4[1]^;*UAS-HvOR13* (n = 11) and response to 10 μg for *Or67d*^GAL4[1]^;UAS-HvOR13* (n = 9). No differences between males and female responses were recorded. High quality figures are available online.

**Figure 3.**
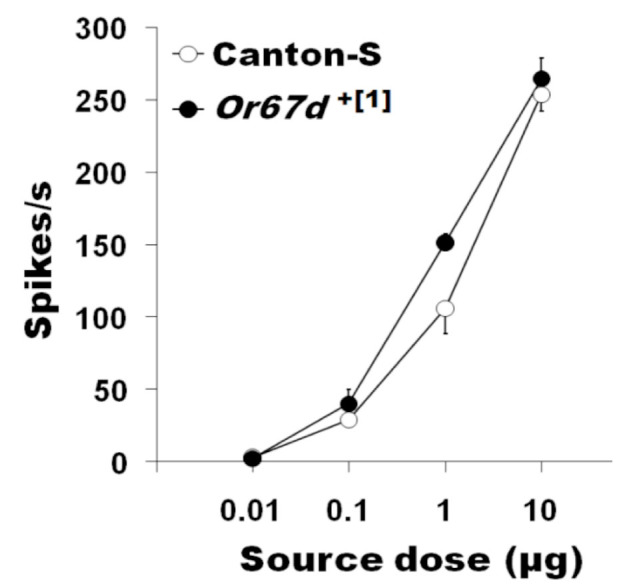
*Drosophila melanogaster* trichoid sensilla respond with high sensitivity to the cVA sex pheromone in Canton S and the Or67d^+[1]^ control line. High quality figures are available online.

**Figure 4.**
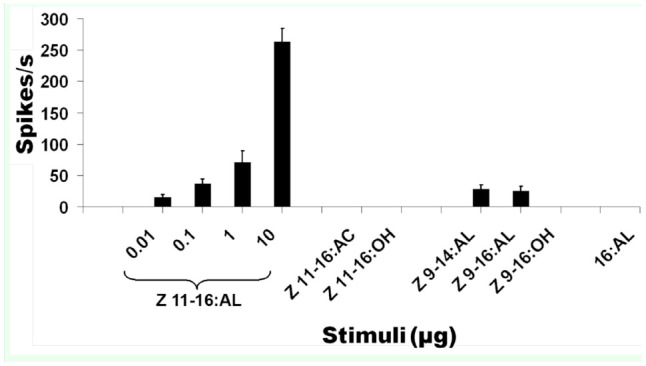
Response spectrum of the pheromone receptor HvORl 3 expressed in an olfactory receptor neuron housed in a trichoid sensillum of *Drosophila melanogaster Or67d*^GAL4^ ^[1]^;*UAS-HvOR13*. All compounds (Z11-l6:Ald, Z9-l4:Ald, Z9-16:Ald, 16:Ald, Z11-16:OAc, Z11-16:OH, and Z9-16:OH) were tested at 10 μg source dose, except Z11-16:Ald, which was tested at 0.01, 0.1, 1, and 10 μg source doses (n = 11).High quality figures are available online.

These experiments were designed to examine whether HvOR13 expressed in *D. melanogaster* Tl sensilla have high specificity and sensitivity for Z11-16:Ald, as compared to Wang et al. (2011). *Or67d*^GAL4 [1]^;UAS-HvOR13 T1 sensilla responded to varying doses (0.01–10 μg) of Z11-16:Ald in a dosedependent manner (Figure 2A, B) while T1 sensilla of *Or67d*^GAL4 [1]^;UAS-HvOR13* responded only to the highest dose of Z11-16:Ald tested (10 μg) (Figure 2B). The control *Or67d*^+[1]^ did not respond to any of the *H. virescens* pheromone components, except for Z11-16:OAc at the highest dose (10 μg) tested (87 ± 15.87 spikes/sec, n = 7), and responded to doses of cVA between 0.1–10 μg (Figure 3). In addition, *Or67d*^GAL4 [1]^;*UAS-HvORl3* Tl sensilla responded only to high doses (10 μg) of Z9-14:Ald and Z9-16:Ald, and no response to 16:Ald, Z11-16:OAc, Z11-16:OH, or Z9-16:OH was observed at the same high dose (Figure 4). *Or67d*^GAL4[1]^;UAS-HvOR13* Tl sensilla did not respond to any of the other pheromone compounds tested. *Or67d*^GAL4 [1]^;*UAS-HvOR15* T1 sensilla did not respond to any of the pheromone compounds tested.

**Table 1.**
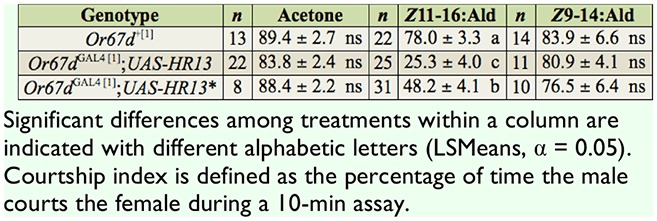
Courtship indices (mean ± SE) for males of the indicated genotypes paired with *Or67d*^+[1]^; virgin female *Drosophila melanogaster* treated with acetone, Z11-16:Ald, or Z9-14:Ald.

### Behavioral assays

In single pair courtship assays, the genotypes *Or67d*^+[1]^; *Or67d*^GAL4 [1]^; *UAS-HvOR13,* and *Or67d*^GAL4 [1]^;UAS-HvORl3* had comparable responses when paired with *Or67d*^+[1]^ virgin female flies treated with acetone (*F*_2,40_ = 1.60, *p* = 0.2152) (Table 1). Comparable responses were also recorded when females were treated with Z9-14:Ald (F_2,32_ = 0.58, *p* = 0.5634), a *H. virescens* pheromone component for which no electrophysiological responses were observed in the transgenic fly strains. In contrast, *Or67d*^GAL4 [1]^*; UAS-HvOR13* and *Or67d*^GAL4 [1]^;UAS-HvORl3* males exhibited a lower courtship index than that of the *Or67d*^+[1]^ males when paired with female flies treated with Z11-16:Ald (*F*_2,44.7_ = 63.67, *p* < 0.0001) (Table 1). The lower courtship index indicates that male flies expressing HvOR13 detected Z11-16:Ald and responded accordingly.

### *HvOR13* expression and cDNA sequence comparison

*HvOR13* mRNA expression levels in *Or67d*^GAL4 [1]^*; UAS-HvOR13 (HvOR13/RP49* = 0.038 ± 0.005) and *Or67d*^GAL4 [1]^;UAS-HvOR13* *(HvOR13/RP49* = 0.043 ± 0.005) flies were examined, but no differences in transcript abundance were found between these lines (*t* = 0.41, *p* = 0.91), indicating that differences in electrophysiological and behavioral responses are not related to differences in gene expression. *HvOR13* coding sequence comparison between *H. virescens* and the *Or67d*^GAL4 [1]^;*UAS-HvOR13,* and *Or67d*^GAL4^^[1]^;UAS-HvOR13* flies indicated some sequence variation that may represent nucleotide polymorphisms (122A>C; 287C>T; 387C>T; 504A>G; 714T>C; 717C>T;732G>A;861T>C) or single point mutations (160G>C; 261T>C;606C>T;801G>A;877A>G; 947T>A;1150C>T). Point mutations led to two conserved amino acid changes at position 54 (V → L) and 293 (M → V), a major amino acid change at position 316 (L → Q), and an early termination codon at position 384 (Q → stop codon) in *Or67d*^GAL4 [1]^; UAS-HR13* flies (Figure 1).

## Discussion

The combined electrophysiological and behavioral data showed that the response of *HvORl3* expressed in *D. melanogaster* T1 sensilla *(OR67d*^GAL4^;*UAS)* was highly sensitive and specific and in agreement with the findings of Syed et al. (2010) for *BmORl* in the same heterologous expression system. Expression of *HvOR13* and other *H. virescens* pheromone receptors in Flp-In T-Rex293/Gα^15^ cells (Grosse-Wilde et al. 2007) indicated that this heterologous expression system is not as specific as the *D. melanogaster* system that we used. Moreover, pheromone binding proteins and an organic solvent had to be used in the cell system to increase sensitivity and specificity of the *H. virescens* receptors tested. It is possible that *D. melanogaster* biochemical components involved in cVA detection may increase the sensitivity and specificity of moth pheromone receptors expressed in the *OR67d^GAL4^*;*UAS* system.

Recently, Wang et al. (2011) expressed HvORl 3 in *X. laevis* oocytes and found a level of specificity of the receptor response based on their electrophysiological results that is comparable to the results reported in our study. In the same study, it was found that HvORl 5 expressed in *X. laevis* oocytes did not respond to *H. virescens* pheromone components and 50 general odorants. Thus, the lack of response of *Or67d*^GAL4 [1]^;*UAS-HvOR15* flies to the seven pheromone components tested in our study supports the findings of Wang et al. (2011). Despite the technical differences between the *D. melanogaster* and the *X. laevis* systems, both are useful for characterization of pheromone receptors in moths and possibly other insect taxa. It is important to note that when *X. laevis* oocytes and *D. melanogaster* empty neurons were used for functional characterization of a large set of *Anopheles gambiae* odorant receptors, the two methods did not always produce similar results (Carey et al. 2010; Wang et al. 2010). This emphasizes the need to conduct both types of assays in order to make firm conclusions about functional specificity of important receptors. In addition, expressing moth odorant receptors in fly trichoids offers a system to test the behavioral output in response to cognate pheromone ligands.

The results of our study also suggest that a point mutation leading to a major amino acid change at position 316 (L → Q), and another resulting in an early termination codon at 384 (Q → stop codon), in *Or67d*^GAL4 [1]^;UAS-HvOR13* flies may have affected HvORl3 structure and function, which could be associated with differences in electrophysiological and behavioral responses observed between *Or67d*^GAL4 [1]^; *UAS-HvOR13* lines. It has been shown that the C-terminal domain of odorant receptors plays a an important role in the formation of the odorant receptor/Orco heteromeric complex (Benton et al. 2006; de Bruyne et al. 2009), and that the three conserved motifs (A, B, and C) within the last 70–90 amino acid residues of this region appear to have major functional importance (Miller and Tu 2008). As shown in Figure 1, *Or67d*^GAL4 [1]^;UAS-HvORl3* flies have an incomplete HvORl3 C-terminal region. It is possible that the lack of motif C and the partial motif B may be affecting heterodimer formation and the localization and stability of HvORl3 in OR neuron dendrites (Benton et al. 2006; de Bruyne et al. 2009). However, HvORl3 response was not completely obliterated, indicating that a C-terminal missing the last 42 amino acids could affect but not necessarily abolish receptor function. Also, the N-terminal half of odorant receptors has been suggested to be involved in odor binding (de Bruyne et al. 2009), which may explain how this mutated pheromone receptor could possibly bind to its ligand, Z11-16:Ald. Thus, we suggest that the *Or67dGAL4-UAS* system is not only a powerful tool for characterization of insect pheromone receptors, but is also very useful for testing mutations that could affect pheromone receptor function.

## Abbreviations

OR,: odorant receptor;
Z9-14:Ald,: (Z)-9-tetradecenal;
Z9-16:Ald,: (Z)-9-hexadecenal;
Z9-16:OH,: (Z)-9-hexadecen-1-ol;
Z11-16:Ald,: (Z)-11-hexadecenal;
Z11-16:OAc,: (Z)-11-hexadecenyl acetate;
Z11-16:OH,: (Z)-11-hexadecen-1-ol
